# The effect of attachment systems and denture cleaning methods on microbial biomass and composition in implant-supported overdentures: an experimental study

**DOI:** 10.1186/s40729-024-00564-9

**Published:** 2024-10-17

**Authors:** Yuwei Zhao, Xin Yang, Bixin Wen, Yuqing Li, Haiyang Yu

**Affiliations:** 1 State Key Laboratory of Oral Diseases, National Clinical Research Center for Oral Disease, West China Hospital of Stomatology, Sichuan University, No. 14, 3rd Section of Ren Min Nan Rd., Chengdu, Sichuan 610041 People’s Republic of China; 2 Department of Prosthodontics, West China Hospital of Stomatology, Sichuan University, Chengdu, People’s Republic of China

**Keywords:** Implant-supported overdenture, Attachment system, Ultrasonic clean, Chemical cleaner solution, Biofilm adhesion

## Abstract

**Objective:**

This research endeavors to scrutinize the influence of attachment systems and denture cleaning methodologies on microbial biomass and composition within the realm of implant-supported overdentures, a crucial consideration for patients with dentition defects necessitating such prosthetic solutions.

**Subjects and methods:**

Employing five polymethyl methacrylate specimens designed to emulate the fitting surfaces of traditional dentures and implant-supported overdentures. Following the polishing of each specimen and the quantification of its roughness, co-cultivation with three distinct microbial strains ensued, culminating in ultrasonic cleaning in water. The bar-clip group, differentiated by the depth of attachment, underwent cleaning employing four diverse methods. Biomass quantities were meticulously recorded both pre and post cleaning interventions, with subsequent data analysis via t-testing and one-way ANOVA, maintaining a significance level of α = 0.05.

**Results:**

The bar-clip groups demonstrated an elevated degree of microbial adhesion, with the deeper locator group exhibiting heightened biomass residue post-cleaning, indicative of increased cleaning complexity. Ultrasonic cleaning predominantly targeted biofilm and deceased bacteria, whereas chemical cleaners primarily reduced the quantity of viable bacteria. The synergistic application of ultrasonics and chemical cleaning treatments yielded the minimal biomass residue.

**Conclusion:**

In contemplating the utilization of dentures milled by dental computer-aided design/manufacturing systems, meticulous pre-use surface polishing is imperative. The extent of biofilm adhesion correlates with the chosen attachment system. This study advocates for the incorporation of ultrasonic cleaning in conjunction with chemical cleaning solutions to optimize the removal of biofilm and live cellular entities in the context of implant-supported overdentures.

## Introduction

Osseointegrated implants have proven a successful and popular treatment for edentulism. Implant-supported overdentures have lower surgical morbidity rates and more acceptable economic costs than implant-fixed dentures because treatment with implant-supported fixed prostheses may be detrimental to the health of elderly patients or those with systemic disease [1]. Compared with traditional removable dentures, implant-supported overdentures exhibit increased retention, support, and stability [2]. Therefore, implant-supported dentures have been proposed as an alternative treatment option for edentulism patients with poor residual ridge conditions.

Implant overdentures and implants are commonly retained using bar, ball, locator, and telescopic crown attachments [3]. These attachments require additional space, and the fitting surface is more complicated as a result. The occlusal space and structure are different for each type of attachment, but regardless of the type, this space becomes a reservoir of microorganisms, increasing the likelihood of bad breath and denture stomatitis [4]. In a 10-year clinical evaluation of implant overdentures, it was found that the accumulation of plaque is related to peri-implantation mucositis and peri-implantitis [5]. Denture plaque contains potential respiratory pathogens, which have been shown to be a threat to patients’ overall health [6].

However, denture wearers are generally elderly and immobile, with a high prevalence of joint inflammation—which results in restricted hand movement—poor vision, and cognitive impairment [7]. It has been found that 36.1% of senior patients in general hospitals and about 23% of elderly people in the community aged 65 years or older have minor cognitive impairments and require the support of carers [8]. Cleaning dentures is often difficult for this group. In addition, the structure of the denture fitting surface will affect the adhesion of microorganisms, complicating the microbial situation of the implanted overdentures, and patients cannot easily or effectively clean the finer structure of the implanted overdentures using manual and flexible brushing [4, 9].

The present study on cleaning methods largely focuses on the research on the plane of the conventional denture base, and cleaning guidelines for implanted overdentures have not been proposed [10, 11]. Therefore, the purpose of this study is to evaluate the effects of different denture attachments and cleaning methods on cleaning efficiency. This study aims to evaluate microbial adhesion for several attachment systems in implant overdentures and the effects of several cleaning methods, which may rapidly and easily reduce microbial adhesion, reduce the occurrence of problems related to overdentures, provide a reference that dentists can use to choose the most appropriate attachment system in a clinical setting, and ultimately preserve the oral health and overall health of wearers.

In this study, polymethyl methacrylate (PMMA) resin disks were milled using computer-aided manufacturing to simulate implant-supported overdentures and conventional dentures.

## Subjects and methods

### Specimen preparation

Based on the vertical height requirements of overdenture attachments in clinical implant-supported overdenture systems, several standard models were designed using the Solidworks software package (Dassault Systems SA, Conker County, MA, America) (Table 1). The model was designed as a 28 mm * 8 mm * 8 mm box that has a surface with an attachment space for a denture fitting surface; for one sample, the surface was left unpolished, and for the other five samples the surface was polished [10]. PMMA model resin (Bio-pink, Aditie, Qinhuangdao, China) was milled using a CAD/CAM milling unit (K5 impression, VHF, Ammerbuch, Germany) according to the model STL files. All samples were sonicated with ultra-filtered water and then immersed in ultra-filtered water for 48 h to remove residual monomers. For each group, the surfaces were polished with sandpaper from 120 to 2000 mesh (Struers, Copenhagen, Denmark) and inspected using a digital caliper. The overall size of each sample and the thickness of the material removed by polishing were controlled to between 0.2–0.3 μm.

**Table 1:** Five group denture fitting surface model by Solidworks

Group	Fitting surface description	Model sketch (28 * 8 * 8 mm)	Specimen
CO	28 * 8 mm plane		
L2	With two cylindrical wells of 4 mm diameter and 2 mm depth		
L4	With two cylindrical wells of 4 mm diameter and 4 mm depth		
B4	With two cylindrical wells of 6 mm diameter and 4 mm depth, connected with a width of 4 mm		
B6	With two cylindrical wells of 6 mm diameter and 6 mm depth, connected with a width of 4 mm		

*CO* conventional overdenture; *L2, L4* with locator attachment at 2,4 mm; *B4, B6* with bar-clip attachment at 4,6 mm

### Profilometry

The surfaces of each sample were examined using scanning electronic microscopy (JSM-IT500LA, JEOL, Tokyo, Japan). Mean roughness (Sa) was measured using a 3D optical profiler system (Rtec instrument, San Jose, CA, USA) with a confocal objective BF-5X and calculated using Gwydion.

### Bacterial strains, and media

*Streptococcus mutans* UA 159 WT strain and its derivatives were maintained in a sterile brain heart infusion (BHI) broth (Oxoid, Hampshire, England, UK), *Staphylococcus aureus* was maintained in BHI broth at 37 °C, and *Candida albicans* was cultured in a yeast extract peptone glucose (YPGA) medium (Oxoid) overnight at 35 °C in an anaerobic incubator. The resulting bacterial suspensions were respectively diluted to concentrations of 1 × 10^6^ for *Streptococcus mutans*, 1 × 10^6^ for *Staphylococcus aureus*, and 1 × 10^5^ for *Candida albicans* colony forming units (CFUs)/mL, with fresh medium available for further use.

### Surface cleaning

Before cleaning, all specimens were gently washed three times with phosphate buffered saline (PBS).

Immersion cleaning protocol (ICP): specimens were soaked in distilled water at 40 °C for 15 min.

Chemical cleaning protocol (CCP): specimens were soaked in denture cleaning tablet solution (Y-kelin, Beijing, China) for 15 min at 40 °C.

Mechanical cleaning protocol (MCP): specimens were washed in an ultrasonic cleaner (40,000 vibration/sec, Guanshibo, Shenzhen, China) with distilled water for 15 min at 40 °C.

Chemical and mechanical combined cleaning protocol (C + MCP): specimens were washed in an ultrasonic cleaner with denture cleaning tablet solution (Y-kelin, Beijing, China) for 15 min at 40 °C.

### Scanning electron microscopy

Scanning electron microscopy was performed for all samples (JSM-IT500LA, JEOL). Biofilms from each sample were fixed with 2.5% glutaraldehyde for 12 h, serially dehydrated in ethanol, and sputter-coated with gold.

### Biofilm formation and biomass quantification

Before being co-cultured with the microorganisms, the samples were sterilised with 75% ethanol solution and ultraviolet light, after which they were incubated for 2 h with artificial saliva and rinsed with PBS. Next, 8 ml of mixed bacteria liquid was incubated with a resin sample in 12-well polystyrene microtiter plates. After 4 h, the medium was removed and fresh BHI culture containing 1% sucrose was placed in an aerobic environment at 37 °C for 24 h for biofilm formation. Thereafter, the broth was removed and the specimens were washed with PBS to remove the non-adherent cells. All adhesions were collected and stained with crystal violet solution at room temperature for 15 min, and the biomass was quantified using a plate reader at 570 nm [12].

### Live cell counting

The biofilm that formed in the fitting surfaces of the specimens was collected, added to a PBS solution, and fully vortexed. This bacterial solution was then serially diluted and spread on BHI, Baird-Parker, and Chromogenic Candida agar plates. After 48 h incubation, the numbers of colony-forming units were counted.

### Biofilm analysis and structural imaging

Mixed biofilms were generated as described above. Live bacteria with intact cell membranes were labeled with SYTO9, and dead bacteria with compromised membranes were labeled with propidium iodide (Invitrogen, Carlsbad, CA, USA). Images of the biofilm were captured using confocal laser scanning microscopy (Olympus FV3000, Tokyo, Japan). The imaging gates were set to 488 nm for SYTO9 and 561 nm for propidium iodide. Each biofilm was scanned at three random positions, optical sections were used for 3D reconstruction of live/dead cell biofilms with Imaris 7.0.0, and the live-/dead-cell biomass of the biofilms was quantified using ImageJ.

### Statistical analysis

All statistical data were tested to establish whether they met the normal distribution and chi-square criteria; if so, a one-way ANOVA or Tukey’s post-hoc test was performed; if not, the Kruskal–Wallis test or Dunn’s multiple comparison test was performed. All statistical tests were two-tailed with a significance level of 0.05. The statistical analyses were conducted using GraphPad Prism 8.2.0.

## Results

### Roughness measurement

The initial assessment revealed no statistically significant differences among the groups in terms of the unpolished fitting surface condition. However, following the application of an identical polishing protocol to each specimen, a notable transformation unfolded. The mean surface roughness value exhibited a substantial decrease, approximately 12 times lower than that observed in the unpolished condition.

**Fig. 1:**
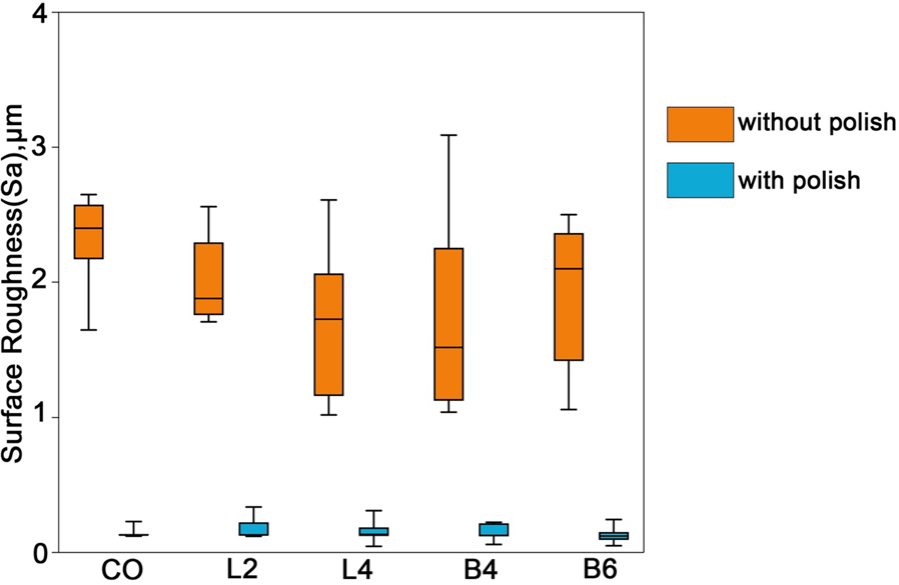
The surface roughness (Sa) of denture specimens. The results are presented as averages +/− standard deviations. CO (n = 5): conventional overdenture; L2 (n = 5), L4 (n = 5): with locator attachment at 2,4 mm; B4 (n = 5), B6 (n = 5): with bar-clip attachment at 4,6 mm. *P* < 0.05

Upon meticulous post-polishing scrutiny, it was determined that there were no statistically significant differences in surface roughness across the various groups. This nuanced analysis, as depicted in Fig. 1, illuminates the detailed comparison of surface roughness among each distinct group. Consequently, a comprehensive understanding emerges, indicating that five distinct overdenture attachment specimens were successfully obtained, showcasing a homogeneity in surface roughness post-polishing, thereby signifying the efficacy of the polishing process in standardizing the surface characteristics across the experimental groups.

### Quantitative analysis of biofilm adhesion and microbial biomass

**Fig. 2:**
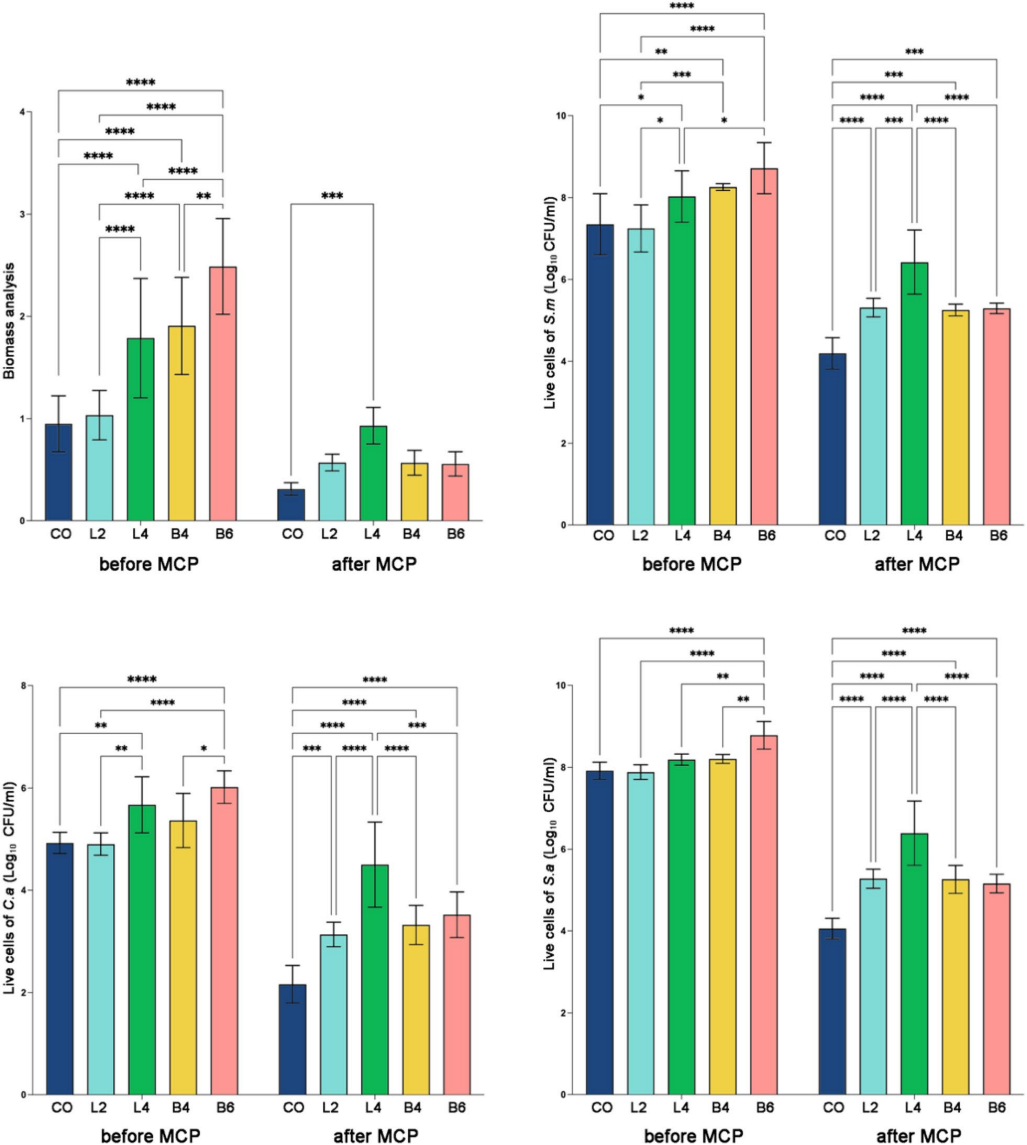
The biomass and live cells of different denture surfaces before and after ultrasonic cleaning. The results are presented as averages +/− standard deviations. CO (n = 5): conventional overdenture; L1 (n = 5), L2 (n = 5): with locator attachment at 2,4 mm; B4 (n = 5), B6 (n = 5): with bar-clip attachment at 4,6 mm. ***, *P* < 0.001; ****, *P* < 0.0001

As illustrated in Fig. 2, prior to undergoing ultrasonic cleaning (MCP), no statistically significant differences were observed in the overall biomass quantity between the CO and L2 groups. It is noteworthy that these two groups displayed the lowest degree of biofilm adhesion. In contrast, the B6 group exhibited the highest degree of biofilm adhesion among the five groups (P < 0.05).

Upon subjecting the samples to ultrasonic cleaning (MCP), distinctive patterns emerged. Specifically, the L4 group presented the largest residual biomass, while the CO group exhibited the smallest residual biomass (P < 0.05). This trend was consistently mirrored in the live cell quantities of each group. Furthermore, a notable inclination towards reduced residual biomass after ultrasonic cleaning (MCP) was evident across all groups.

The data from Fig. 2 indicate that, initially, CO and L2 groups showcased comparable overall biomass, yet with minimal biofilm adhesion. Conversely, B6 exhibited heightened biofilm adhesion. Post-ultrasonic cleaning (MCP), distinctive variations in residual biomass were observed, underscoring the influence of the cleaning process on biofilm adhesion dynamics and live cell quantities across the experimental groups.

### Live/dead microbial ratios for residual biofilm

In the exploration of diverse cleaning protocols, the investigation focused on the L4 group, unraveling intriguing insights. Specifically, the application of C + MCP (specimens subjected to ultrasonic cleaning with a denture cleaning tablet solution) yielded the most promising results, showcasing the lowest residual biomass. Remarkably, this outcome was 2.78 times lower than that observed with CCP (specimens immersed in a denture cleaning tablet solution) and 1.27 times lower than MCP (specimens washed in an ultrasonic cleaner).

Delving deeper into the assessment of live cell quantities and live/dead biofilm ratios, ICP (specimens soaked in distilled water) emerged as the protocol with the highest ratio. In contrast, C + MCP (specimens washed in an ultrasonic cleaner with a denture cleaning tablet solution) demonstrated the lowest ratio. It is noteworthy that CCP (specimens immersed in a denture cleaning tablet solution) exhibited notable efficacy in removing live bacteria, while MCP (specimens washed in an ultrasonic cleaner) demonstrated a superior capacity for eliminating dead bacteria.

**Fig. 3:**
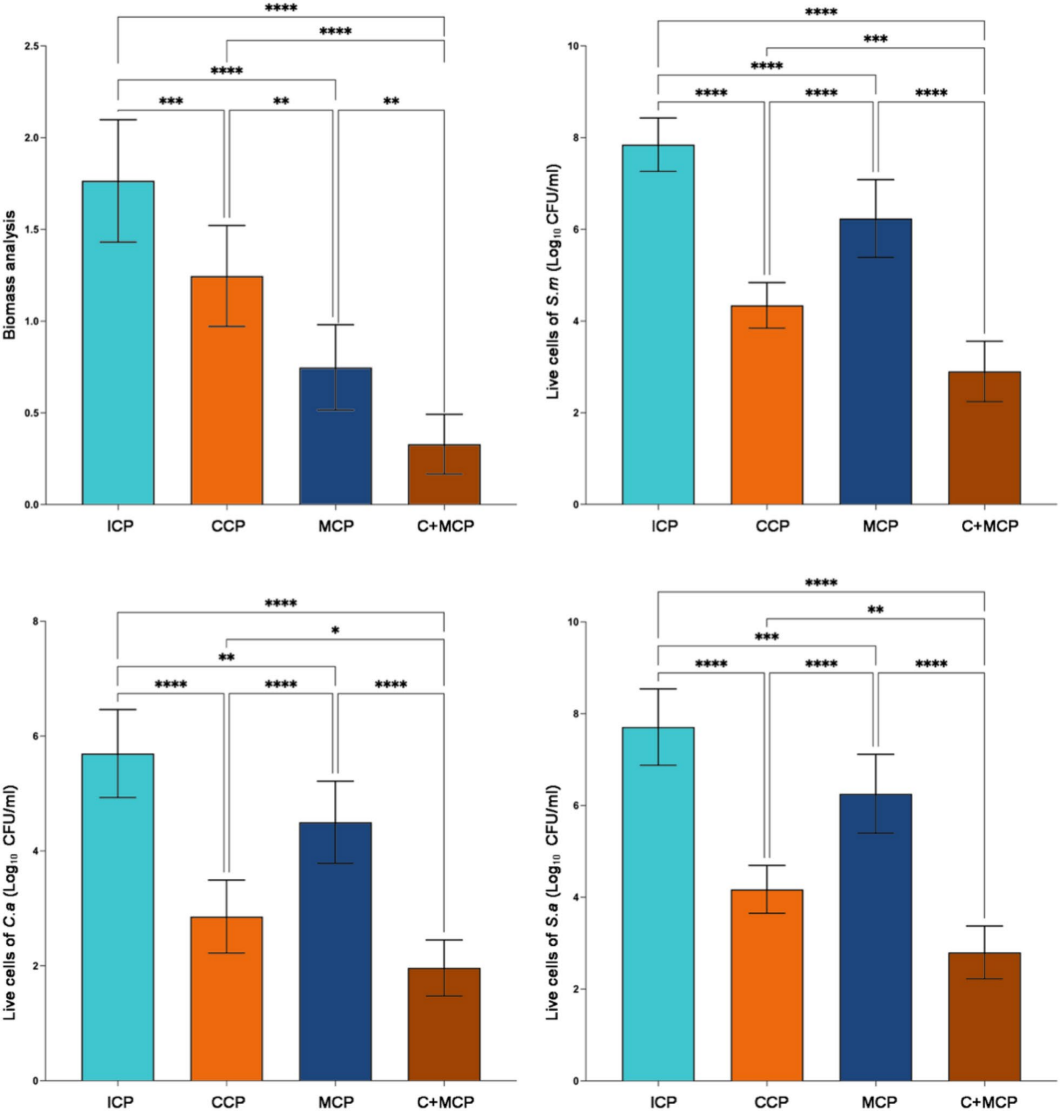
The biomass and live cells of L4 after four cleaning methods. The results are presented as averages +/− standard deviations. L4: with locator attachment at 4 mm. ICP (n = 5): Immersion cleaning protocol. CCP (n = 5): chemical cleaning protocol. MCP (n = 5): Mechanical cleaning protocol. C + MCP (n = 5): Chemical and mechanical combined cleaning protocol. **, *P* < 0.01; ***, *P* < 0.001; ****, *P* < 0.0001

**Fig. 4:**
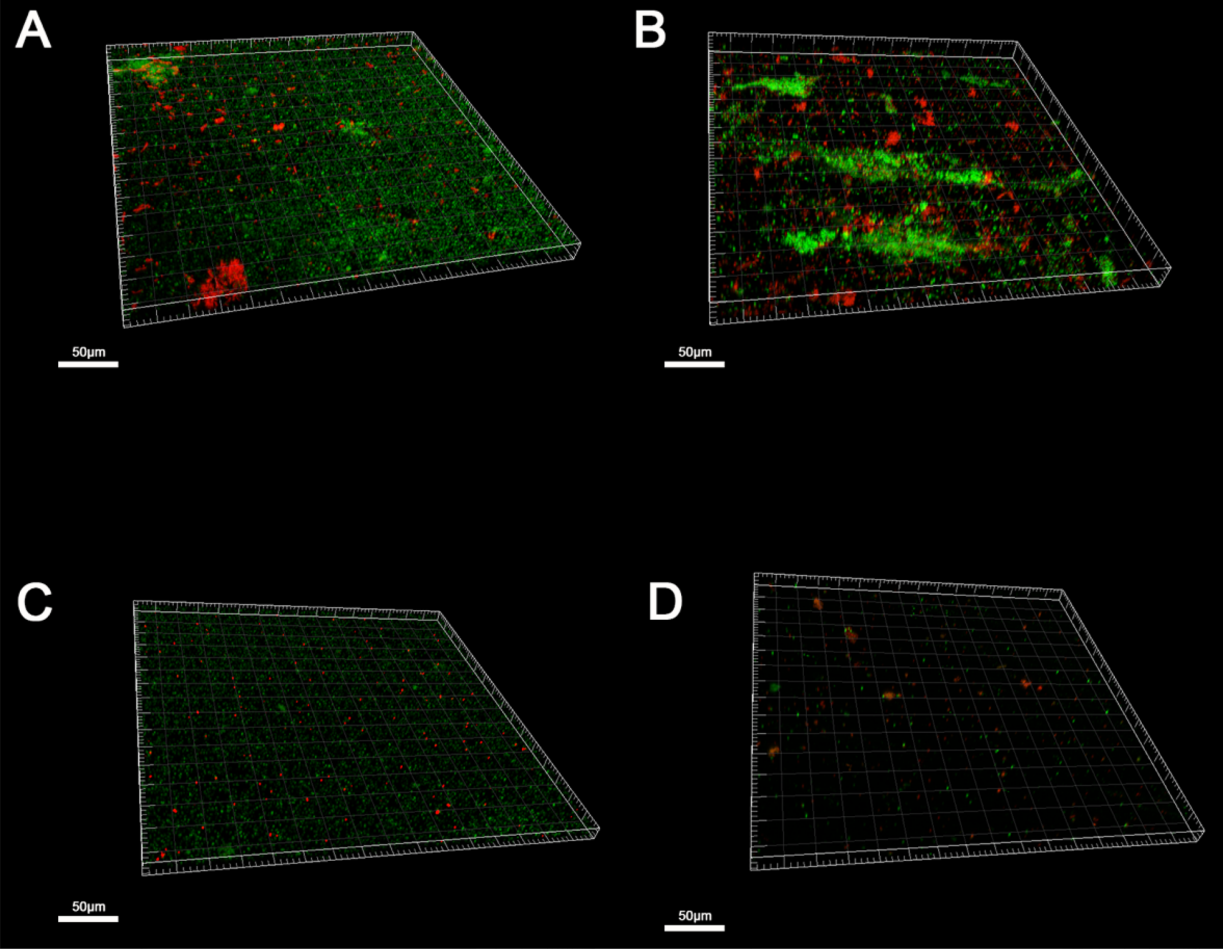
Live and dead bacterial cells of the biofilm of L4 after four cleaning methods were labeled with SYTO9 and PI (Invitrogen; green, live cells; red, dead cells. Images were taken at 40× magnification. Three-dimensional reconstruction of the biofilms of live/dead cells was performed with Imaris 7.0.0.A. with ICP B. with CCP C. with MCP D. with C + MCP. L4: with locator attachment at 4 mm. *ICP* immersion cleaning protocol, *CCP* chemical cleaning protocol, *MCP* mechanical cleaning protocol, *C + MCP* chemical and mechanical combined cleaning protocol

These nuanced findings, meticulously presented in Figs. 3, 4 and detailed in Table 2, contribute a comprehensive understanding of the distinct impacts of various cleaning protocols on parameters such as residual biomass, live cell quantities, and biofilm ratios within the experimental framework. This comprehensive analysis serves to elucidate the differential effectiveness of cleaning strategies in shaping the biofilm dynamics and microbial composition in the L4 group.

**Table 2:** The effect of different cleaning protocols is presented as residual biomass quantities (OD_570_) and live/dead cell ration on L4 group (mean +/− standard deviations)

Cleaning protocol	Residual Biomass quantities	Live/dead cell ratio
ICP	1.77 ± 0.33	22.03 ± 7.45
CCP	1.25 ± 0.28	1.81 ± 0.57
MCP	0.75 ± 0.23	7.20 ± 2.74
C + MCP	0.33 ± 0.16	0.42 ± 0.22

L4: with locator attachment at 4 mm. ICP (n = 5): immersion cleaning protocol. CCP (n = 5): chemical cleaning protocol. MCP (n = 5): mechanical cleaning protocol. C + MCP (n = 5): chemical and mechanical combined cleaning protocol

## Discussion

CAD/CAM PMMA resin has comparable biocompatibility and superior mechanical qualities to standard thermosetting resins because of the high pressures used in the polymerisation process during their manufacture [13]. CAD/CAM PMMA is generally deemed superior to conventionally processed PMMA for the fabrication of complete dentures [14]. The specimens used in this study were milled using the same dental CAD/CAM system to eliminate errors caused by differences in the fabrication process. Previous studies have found that surfaces manufactured by CAD/CAM show undulating ripples due to the use of different milling machines, milling burrs and burr actions. However, these surfaces still have smoother and more hydrophilic surfaces than conventional dentures made from heat-polymerising resin [15]. Specimens manufactured by the same machine show a high degree of consistency. Another study showed that CAD-CAM-milled and rapidly prototyped complete denture resins performed similarly in terms of surface roughness, but the milled denture resins have superior mechanical properties and eliminate manufacturing differences [16].

It has been reported that there is a relationship between denture surface roughness and bacterial adhesion. In our study, the roughness of resin blocks was reduced about 12 times after polishing [17, 18]. The roughness of the polished surface of the samples was 0.15 ± 0.06 µm, which is close to the specified threshold value of 0.2 µm for microbial adhesion and plaque retention of restorative dental materials [19]. It will also improve the patient’s wearing experience. The overdenture fabricated by CAD/CAM should be finely polished before use in the clinic to guarantee their cleanability for elderly wearers. However, to enable fitting to the oral mucosa, the fitting surface usually cannot be polished in clinic.

In this study, the three-strain biofilms used to simulate the denture plaque consisted of *Streptococcus mutans*, *Staphylococcus aureus*, and *Candida albicans*. Denture plaque is formed as a result of the participation of a variety of microorganisms in the mouth, and these three species are representative of the intraoral multi-species biofilm environment [20–23]. Among them, *Streptococcus mutans* is an oral commensal bacteria species which can metabolise sugars and synthesise water-insoluble polysaccharides (WIS-EPS) [24]. WIS-EPS are known to be an important component of biofilm formation and adhesion [25, 26]. *Candida albicans*, on the other hand, is an important causative agent of denture stomatitis, a disease commonly seen in elderly people who wear dentures for long periods [27, 28]. In this mixed biofilm, the extracellular matrix could improve the adhesion of microorganisms and their tolerance to antimicrobial agents, and these structures maintained the mechanical stability of the biofilm [26]. This oral biofilm model reduces complexity but maintains an adequate degree of similarity to real-life conditions.

For bar-overdentures, a gap is required for a hygiene space under the bar, the thickness of the bar and the clip and housing. More space is often required compared to locator attachments [29]. These two kinds of attachment systems are usually used to compensate for the angle of the implant [30]. Furthermore, the deeper attachment space of overdentures is often designed to correspond to a higher bar in the oral cavity, which is decided upon according to the vertical occlusal dimension (VOD) for each patient.

A previous study showed that ultrasonic cleaning was as efficient as brushing, and it was used in this study as it reduces the effects of human error [31]. Even though brushing is generally considered to be easy, and most denture wearers use brushing to clean their dentures, brushing with dentifrice may damage the surface of the dentures and reduce the retention of attachments [32]. Ultrasonic cleaning machines are often used for fine cleaning and removing soil from joints and other areas that are too difficult to clean using other methods. In the ultrasonic field, the biofilm will be disturbed by collapsing and releasing shear forces due to cavitation [33, 34]. This method has been used to clean dentures and implants without causing damage [35]. It is recommended that dentures are cleaned with warm water [36]. Of the approaches examined here, the total amount of biofilm on the fitting surface of the deeper locator attachment overdenture was highest after ultrasonic cleaning. This may be due to the deep and narrow space of the fitting surface—cavitation bubbles tend to be weaker in spaces of this type [35].

Regarding the four methods used to clean the L4 group, the immersed cleaning protocol generally removed WIS-EPS and environmental DNA (eDNA), including some proteins produced by microbes in biofilm [37]. Commercial denture cleaners usually consist of sodium bicarbonate, hydrogen peroxide, and oxygen gas; this kind of cleaner provides cleaning and bleaching via hyperthermic softening, alkaline saponification, cavitation loosening, dissolution, and oxidative decolorisation of various biological masses. This enables the biofilm structure of denture surfaces to be broken, thus destroying the live bacteria [38]. In a study of nursing home residents, it was revealed that the frequency of use of denture cleansers was negatively correlated with the number of *Candida *spp*.* present. In the study, denture plaques were collected by volunteers and cultured to identify *Candida *spp*.* It was difficult to exclude the impact of the dentures themselves, such as usage time or manufacturing method [39]. When it comes to implant-supported dentures, the situation is more complex. However, in our study, the complicated fitting surface of implant-supported overdentures was simplified to a model which can be manufactured and using by the same procedure. Furthermore, it has been revealed that the attachments of implant-supported dentures tend to lose their retention when soaked in sodium hypochlorite, another oxide that can be used to keep dentures clean [40]. Therefore, sodium bicarbonate is recommended over sodium hypochlorite for cleaning implant-supported dentures and is widely used in commercial denture cleaners. The live/dead ratio and biofilm imaging may reveal the differences between chemical cleaning and mechanical cleaning.

The present study does have some limits: a three-strain biofilm is different from the complex oral microecological environment, due to the effects of temperature, eating, saliva, and individual differences among patients. The surfaces of dentures are also much more irregular than these specimens. Further study is needed to reveal the effects of these factors.

## Conclusions

Within the limitations of this in vitro study, we drew the following conclusions. More microbes will adhere to dentures with a bar-clip attachment than overdentures with locator attachments. However, a larger quantity of microbe residue was observed in dentures with locator attachments. For elderly patients who use implant-supported overdentures, a combination of ultrasonic cleaning and a chemical cleaning solution is recommended to achieve effective cleaning.

## Data Availability

No datasets were generated or analysed during the current study.

## Abbreviations

PMMA: Polymethyl methacrylate
ICP: Immersion cleaning protocol
CCP: Chemical cleaning protocol
MCP: Mechanical cleaning protocol
C + MCP: Chemical and mechanical combined cleaning protocol
